# Variable high-contrast all-optical self-switching of femtosecond solitonic pulses in multi-dual-core optical fiber with distributed optical asymmetry

**DOI:** 10.1007/s11082-026-09092-9

**Published:** 2026-08-29

**Authors:** Mateusz Winkowski, Mattia Longobucco, Jozef Chovan, Dariusz Pysz, Ryszard Buczynski, Ignác Bugár

**Affiliations:** 1 Łukasiewicz Research Network, Institute of Microelectronics and Photonics, Aleja Lotników 32/46, Warsaw, 02-668 Poland; 2 Faculty of Physics, University of Warsaw, Ludwika Pasteura 5, Warsaw, 02-093 Poland; 3 Slovak Centre of Scientific and Technical Information, International Laser Centre, Ilkovičova 3, Bratislava, 84104 Slovakia; 4 Photonics Institute, TU Wien, Gusshausstrasse 27/387, Vienna, 1040 Austria; 5 Institute of Chemistry and Environmental Sciences, University of Ss. Cyril and Metodius, Námestie J. Herdu 577/2, Trnava, 917 01 Slovakia

**Keywords:** Nonlinear fiber coupler, All-optical switching, Multi-core fiber, Soliton self-trapping, Dual-core optical asymmetry

## Abstract

Effective alloptical selfswitching of Cband femtosecond pulses, driven by pulse energy in a nonlinear fiber coupler, was experimentally investigated. A key contribution of this work is the detailed analysis of optical asymmetry between the two fiber cores influencing the switching mechanism. The asymmetry was examined using a multicore, highly nonlinear softglass fiber containing five noninteracting dualcore units with distinct propagationconstant mismatches. The study systematically evaluated the fiber length and input polarization effects, tracking changes in the output intensity distribution and extending the analysis to the spectral domain. Different regimes of highcontrast switching based on soliton selftrapping were observed and correlated with the degree of optical asymmetry. Beyond its fundamental insights, this work advances the state of the art by reducing the required switching energy, shortening the fiber length, and increasing the number of achievable switching steps.

## Introduction

All-optical signal processing is an intensively investigated domain of applied photonic research (Willner et al. 2014) due to the strong demand in areas of optical logic gates (Volz et al. 2012; Zhou et al. 2024), high bitrate signal transfer (Miao et al. 2016) and optical quantum computing (Zhu et al. 2026). Various mechanisms have been explored to support ultra-fast all-optical signal processing, including coherent interactions in metamaterials (Papaioannou et al. 2016), enhanced nonlinearities in photonic crystal nanocavities (Nozaki et al. 2010), and graphene-loaded plasmonic waveguides (Ono et al. 2020). Although these systems provide high-speed, compactness, and low-energy operation, their complexity limits practical deployment. In contrast, the concept of Kerr nonlinearity-based nonlinear directional coupler is one of the simplest approaches that enable ultrafast signal processing with the potential to realize a Tb/s compatible switching device. Therefore, it has been studied by numerous theoretical and experimental works in the form of dual-core optical fiber (DCF) since 1982, due to its compatibility with already mature fiber-based optical communication systems (Jensen 1982; Fraga et al. 2006; Rezaei et al. 2022; Andrianov et al. 2025; Richardson et al. 2025).

The early work soon identified that Kerr nonlinearity, which governs the all-optical switching functionality, causes inevitable side effects in the temporal and spectral domain, compromising the pulse-carrying integrity of the information (Liu et al. 2010; Betlej et al. 2006). However, it is possible to prevent such optical signal deterioration in the case of soliton-like propagation, which approach motivated many scientific works analyzing the propagation of various types of solitons in optical couplers (Trillo et al. 1988; Malomed 2019; Younas et al. 2024). The extensive numerical analysis of the problem based on the coupled nonlinear Schrödinger equation revealed an advantageous regime that allows high-contrast switching, based on self-trapping of a high-order soliton (Stajanca and Bugar 2016). This process is based on the self-compression of the input pulse, which causes significant enhancement of the peak power and simultaneously the Kerr nonlinearity effect. The dramatic increase of the effective refractive index in the core, where the self-compression is localized, creates strong dual-core asymmetry, which prevents further field transfer to the second core. After the soliton compression point the high-order soliton breaks apart to more pulses and generates often dispersion waves, however, at proper settings of the dispersion/nonlinear properties one post-pulse became dominant bearing the majority of the original pulse energy (Longobucco et al. 2019, 2020a, b).

In this case, the cross-core transfer is nonlinearly prevented for longer propagation distance due to the preservation of such post-pulse width and energy, thus it is trapped in the core where occurred. Beyond the fundamental insight acquired during the analysis of the problem, the advantage of the self-trapping process is the following: the core, where the compressed pulse occurs, is exchangeable by the input pulse energy. At lower energies, the pulse can transfer from the excited to the cross core and traps afterwards, however at sufficient higher energy it is compressed and trapped already in the excited core, because the soliton compression distance reduces with increasing energy (Agrawal 2008). Such a special property of the dual-core high-order solitonic propagation allows the achievement of high switching contrasts beyond 10 dB, which was hardly accessible in other regimes of such ultrafast nonlinear interaction. The exchangeable self-trapping regime has been identified in the frame of theoretical study of the dual-core soliton like femtosecond propagation in highly nonlinear photonic crystal fiber. In this case, the switching between the cores is controlled by the pulse energy in the sub100 pJ range, which is a further advantageous property of the proposed soliton self-trapping approach (Stajanca and Bugar 2016).

Later, photonic crystal fiber technology was exchanged by the simple cladding two-component soft glass fiber design keeping the highly nonlinear and high-index contrast character required for the proposed low-energy solitonic switching performance (Cimek et al. 2016). This technology has brought experimental results demonstrating reversible self-switching of femtosecond pulses (Longobucco et al. 2020a, b). The dedicated numerical simulations have confirmed the role of the soliton self-trapping phenomena behind this achievement, considering a totally symmetrical dual-core structure (Nguyen et al. 2020). However, the follow-up experimental-theoretical study comparing the switching performance under subsequent excitation of the two fiber cores has revealed, that the asymmetry of the novel DCF is not negligible (Nguyen et al. 2023). Moreover, it can help establish robust switching performance by setting proper fiber and exciting pulse parameters. It has been shown that the moderate mismatch between the effective refractive index of the two cores ensures stable output field dominance at the first or second core despite of small fluctuation of the input pulse energy, in correspondence with other theoretical studies (Uthayakumar et al. 2013). In contrast, the totally symmetrical structure expresses a higher sensitivity to energy fluctuations, thus the redirection of the signal between the two output ports is less robust. The experimental results partially confirmed this concept by analyzing only one DCF sample, at which moderate asymmetry was ensured by applying excitation at 1700 nm, as the asymmetry is reducing with wavelength elongation (Curilla et al. 2018).

In this paper, we present an advanced study of the asymmetry effect analyzing a two-component highly nonlinear fiber comprising 5 dual-core units, which does not interact between each other. The complex investigation addressing the all units enables to differentiate between the high- and low-asymmetry character of the high-contrast switching performance. The observations are interpreted in terms of controllable self-trapping of high-order solitons modified by the coupling effect with asymmetric character determined by the geometry of the dual-core structure. Besides the fundamental insights supported also by numerical simulations of the linear optical fiber properties, both the high- and low-asymmetry switching regimes brought improved switching parameters thanks to the comprehensive investigation.

## Specialty nonlinear fiber

High index contrast all-solid fiber made of special soft glasses comprising five individual dual core units (DCU) with distance between the centers of the all core pairs of about 3.1 μm was used. Thermally matched lead-silicate (PBG-08) and borosilicate (UV-710) glasses were used for the fiber cores and for the first cladding, respectively. The main advantages of this glass pair are the extremely high refractive index contrast of ~ 0.4 in the near-infrared region and high non-linearity of the PBG-08 guiding glass that expresses *n*_2_ = 4.3 10^−19^ m^2^/W. Special multicomponent glasses as well as the nonconventional fiber structure were developed and produced entirely in Ł-IMiF. Despite of uniform geometry of the all DCUs made of uniform diameter glass rods, they represent slight variation of the dual-core optical asymmetry as natural effect of the stack-and-draw fabrication process. It originates from the nanometer scale differences between the shape of the two cores and it is quantified as propagation constant mismatch calculated separately for the both cores. The first cladding of the fiber forms a hexagonal close packed structure with 13 elements on the hexagon diagonal. At one corner of the hexagon, the first UV-710 cladding glass rod was exchanged to the PBG08 rod in the preform. Such modification caused during the drawing process homogeneous mergence of this corner with the second cladding made of the same PBG08 glass during the drawing process. By this method, a marker was created that allows one to identify the individual DCUs regardless of the orientation of the central dual-core axis. The compactness of such non-conventional structure with 5 independent coupled waveguides comes from the feature that the one rod distance (~ 1.55 μm) between the two cores allows strong optical coupling between them. While the two rod distance (~ 3.1 μm) already prevents the interaction between the neighbor units in the studied short fiber length ranges below 40 cm. Analyzing the nonlinear response of all 5 units, it was possible to differentiate between them as the switching performance and optimal fiber length are highly sensitive on the dual-core optical symmetry varying among the units.

## Experimental setup and measurement methods

**Fig. 1:**
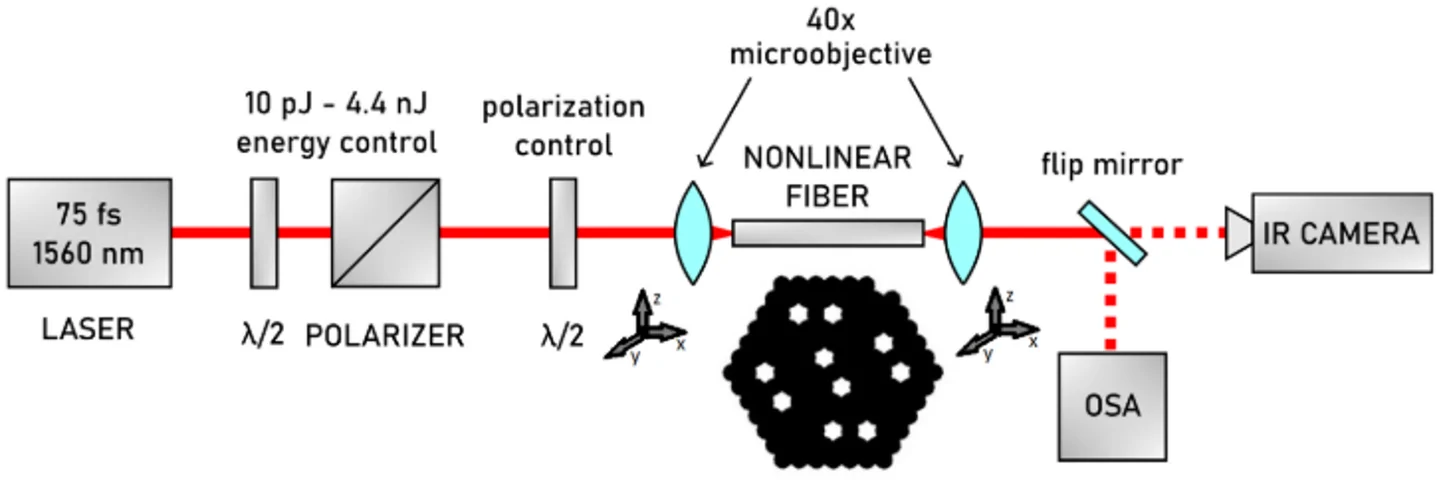
Simplified experimental setup together with the cross section of the 10-core nonlinear fiber. Under marker orientation in the upper right position the individual dual-core unit are labeled as top/bottom/left/right/central

The experimental setup is presented in the Fig. 1. Menlo CFiber femtosecond laser was used as ultrashort light source generating 1560 nm pulses with maximal 4.5 nJ energy and width of about 75 fs at repetition rate of 100 MHz. A microobjective with magnification of 40x was used to in-couple the laser beam sequentially to each fiber core. The nonlinear response of the excited core was analyzed as the intensity ratio registered at the two output ports of the respective unit based on the input pulse energy separately for the two main polarization directions. The pulse energy was controlled by rotation of a half-wave plate placed before a polarizer in the range of 10 pJ - 4.4 nJ. Sequentially, the second half-wave plate set the polarization parallel or perpendicular to the dual-core axis (crossing the two core centers) after the polarizer. Separate data acquisition for the both polarization directions was realized due to the inherent birefringence of coupled waveguides, causing different optical properties for parallel and perpendicular polarization settings. The output from the DCUs, outcoupled by a second 40x microobjective, was monitored in the image plane of the fiber facet by an IR camera (Ophir-Spiricon SP90419) capturing always just the two cores of the excited unit. Additionally, the individual cores of the unit were monitored by an optical spectrum analyzer (Yokogawa AQ6375B) placed after a flip-mirror ensuring the redirection of the output field from the camera to the collecting fiber of the spectrometer. By this way, camera image and spectral pair series were registered depending on the input pulse energy, excited core selection, and polarization direction. Furthermore, the nonlinear response of all dual-core units were examined in the couple centimeter region expecting the characteristic distances as coupling length and soliton compression point in the mm range. The whole registration procedure was repeated for ten various fiber lengths. Cutting back the fiber, all 10 cores were subsequently excited and the input energy *E*_in_ dependence of the dual-core extinction ratio:

$$\:\mathrm{E}\mathrm{R}\left({E}_{\mathrm{i}\mathrm{n}}\right)=10\cdot\:\:{\mathrm{log}}_{10}\frac{{E}_{excited}}{{E}_{nonexcited}}\mathrm{i}\mathrm{n}\:\mathrm{d}\mathrm{B},$$

where *E*_*xxx*_ is the output field energy in the corresponding cores of the excited unit, was calculated processing both the camera and spectral results. In all cases, four measurement data were averaged before further processing. The camera exposure time varied from 400 to 1 ms in order to utilize full dynamic range of the device while working with different pulse energies.

## Data processing and numerical calculations

In the context of the experimental study described above, when the output ER value changed its sign (dominance exchange) under monotonous input pulse energy enhancement, output field registration took place. Due to the JPG format limitations (8-bit data depth), some information from a 12-bit IR camera would be lost. Thus, the data was stored in the numerical version (CSV file), utilizing the full native dynamic range of the detector. Afterwards, it was processed using Python programming language. To ensure uniformity, the data processing procedure was automated starting with background subtraction using the ‘dark file’ captured at closed laser shutter. Afterward, median and moving average filtering was applied to eliminate camera artifacts – so called hot pixels, and smooth the spatial intensity profile. Then, the algorithm automatically finds the cores and integrates their output energies over a uniform number of pixels. Finally, the dual-core extinction ratio value was calculated.

In the case of the spectral results, optical spectrum analyzer provided data related to both cores of the studied unit. Properly positioned iris aperture was used to register field originating exclusively from one core. The aperture was realigned after one energy series collection to the second core, preserving its diameter, and subsequently the second series was registered. Then, the spectral profile of the input energy dependent ER was calculated as:

$$\:ER({E}_{in},\lambda\:)=10\cdot\:\:{{log}}_{10}\frac{{I}_{excited}\left(\lambda\:\right)}{{I}_{nonexcited}\left(\lambda\:\right)}$$

using the smoothed original spectra. Spectral data processing was also fully automated. The multicore fiber samples linear optical properties were numerically analyzed by a commercial Ansys Lumerical mode solver based on the finite difference time domain method. The cross section of the used sample was imaged by scanning electron microscope with a resolution allowing separate evaluation of the all dual-core units (2.46 × 2.46 nm). The image was binarized and imported into the solver approximating the two glasses used by Sellmeier coefficients acquired by measuring bulk glass samples. Spectral characteristics of quantities such as propagation constant, effective index of refraction, group velocity, and dispersion were calculated related to the fundamental modes of the both cores and the dual-core structure. The output parameters were used to estimate the asymmetry as the difference between propagation constants calculated for the two interacting cores at 1560 nm in the case of the DCUs expressing the best switching performances.

## Overview of the complex self-switching study

In order to identify the dual-core asymmetry level of the DCUs and their potential for effective all-optical switching, complex analysis was accomplished, exciting all 10 cores with increasing pulse energy at the all studied fiber lengths. The units are labeled as central, left, right, top, bottom using orientation presented in Fig.1 inset, where the marker is in the right-top corner of the hexagonal structure. The findings together with the switching performance parameters are summarized in Tables 1 and 2, listing all DCUs and all investigated fiber lengths and also differentiating between the two input orthogonal polarization directions. It is worth mentioning that the laboratory frame angles of the parallel and perpendicular polarizations were different in the case of two set of DCUs: top, bottom vs. left, central, right. Due to different orientations of their dual core axis in the hexagonal structure the parallel/perpendicular polarization direction between the two sets forms an angle of about 60 °. The investigation was carried out for 10 various fiber lengths from 38 to 11 mm.

**Table 1:** Summary of switching performance parameters in fiber length range of 38–29 mm in the case of central/left/right DCUs with identical dual-core axis orientation

Length				
Unit	38mm	36.5mm	31mm	29mm
Central perpendicular	14.1 dB; -1.4 dB	10.0 dB; -2.5 dB	10.3 dB; 2.1 dB	16.8 dB; 5.2 dB
	15.5 dB; 0.54 nJ; 3	12.5 dB; 0.64 nJ; 3	w/o exchange	w/o exchange
Central parallel	3.3 dB; -7.1 dB	6.9 dB; -8.2 dB	10.4 dB; -5.3 dB	8.5 dB; -0.1 dB
	10.4 dB; 0.27 nJ; 3	15.1 dB; 0.19 nJ; 3	15.7 dB; 0.16 nJ; 1	8.6 dB; 4.4 nJ; 2
Right perpendicular	15.3 dB; -1.3 dB	15.1 dB; 0.4 dB	w/o exchange	w/o exchange
	16.6 dB; 2.59 nJ; 3	w/o exchange		
Right parallel	15.1 dB; -1.7 dB	12.5 dB; -0.2 dB	w/o exchange	w/o exchange
	16.8 dB; 2.59 nJ; 1	12.7 dB; 1.15 nJ; 2		
Left perpendicular	19.1 dB; -0.2 dB	14.3 dB; 0.1 dB	w/o exchange	w/o exchange
	19.3 dB; 3.18 nJ; 2	w/o exchange		
Left parallel	19.9 dB; 0.3 dB	7.3 dB; 0.8 dB	w/o exchange	w/o exchange
	w/o exchange	w/o exchange		

Upper values: minimum and maximum ER values of the energy dependent series. Bottom values: switching contrast, switching energy, number of switching steps.

**Table 2:** Summary of switching performance parameters at fiber length range of 29–11 mm in the case of bottom DCU

Length							
Unit	29mm	27mm	25.5mm	18.5mm	16mm	13.7mm	11mm
Bottom perpendicular	17.4 dB; 3.3 dB	16.3 dB; -1.8 dB	6.4 dB; -3.9 dB	4.6 dB; -1.2 dB	w/o exchange	w/o exchange	w/o exchange
	w/o exchange	18.1 dB; 1.15 nJ; 3	10.3 dB; 0.89 nJ; 4	5.8 dB; 4.33 nJ; 2			
Bottom parallel	17.1 dB; 2.4 dB	18.4 dB; -2.5 dB	6.5 dB; -3.2 dB	5.9 dB; -1.6 dB	3.8; -5.0 dB	2.9; -2.2 dB	3.6; -1.2 dB
	w/o exchange	20.9 dB; 1.15 nJ; 3	9.7 dB; 2.28 nJ; 4	7.5 dB; 4.05 nJ; 3	8.8 dB; 0.44 nJ; 1	5.1 dB; 4.4 nJ; 1	4.8 dB; 2.28 nJ; 2

Upper values: minimum and maximum ER values of the energy dependent series. Bottom values: switching contrast, switching energy, number of switching steps.

The tables contain quantitative evaluation of the energy-dependent series, when dominance exchange between the intensities registered at the two output ports took place at the distinct DCU, fiber length, and polarization. Data such as maximum and minimum ER values, switching contrast (SC = ER_max_ – ER_min_), switching energy, and switching steps (number of dominance exchange between the cores) are presented in such cases. Due to the measurement uncertainty we assume the dominance exchange to happen when the ER value changes the sign for two consecutive measurement points and its absolute value exceeds 0.3 dB. If no dominance exchange was observed under the distinct conditions, the corresponding cell is labeled by the phrase “w/o exchange”. Due to the high amount of data required for the complex study, in these cases neither the camera nor the spectral registration took place. To save space and increase the legibility of the presentation, numerous combinations in terms of fiber lengths and specific DCUs are not presented in the tables when no dominance exchange took place. In general, the central, right, and left DCUs expressed switching performance at fiber lengths in range of 38–29 mm and the bottom DCU in range of 29–11 mm; therefore, Tables 1 and 2 are devoted to these two ranges, respectively, to increase even more the legibility. The top DCU expressed any dominance exchange, therefore it is not presented in the tables.

The rich switching possibilities presented in the tables provide a completely new insight of the studied soliton self-switching phenomena both from fundamental and application aspects. First of all, it enables direct comparison of switching performance of more fiber couplers optimized for such functionality with similar geometry, however, by various levels of dual-core asymmetry originating from nanometer scale differences between the shape of the two cores. In fact, the 5 DCUs represent five separate couplers and some of them express excellent switching performance not accessible in the previous versions of this type of DCFs. The dual-core asymmetry affecting the switching performance was addressed in work (Nguyen et al. 2023), however, only from a theoretical point of view, because only one fiber coupler was analyzed. Furthermore, the necessary low asymmetry effect was ensured at the excitation wavelength of 1700 nm, due to the decreasing propagation constant mismatch between the two cores with an increasing wavelength expressing value of = 880 m^−1^. However, significantly lower *δ* was enabled now in the case of bottom perpendicular excitation with a value of 530 m^−1^ at the more important shorter 1560 nm wavelength achieved by the new fiber drawing round. The above-mentioned mismatches were estimated in the frame of numerical calculations analyzing the SEM image of the DCFs cross sections. The complex analysis resulted in propagation mismatch varying in a wide range of 500–2000 m^−1^ across different DCUs considering both polarization directions. An especially advantageous level was identified in the case of central parallel excitation expressing = 2100 m^−1^, which supported the significant improvement of the self-switching approach in terms of switching energy and switching contrast.

The theoretical description of the solitonic femtosecond self-switching process in slightly asymmetric fiber couplers was elaborated in our earlier works, here we provide just a short overview as basic for the interpretation of the new results. The most important mechanism enabling the high-contrast switching is the self-compression phenomena of the high-order solitons, which trap the majority of the pulse energy in one of the cores due to the induced dual-core asymmetry. The soliton compression distance at given fiber parameters and pulse width decreases with increasing pulse energy and the crucial energy level is, which ensures a comparable soliton compression distance and coupling length (Stajanca and Bugar 2016; Longobucco et al. 2019, 2020a, b). Around this energy level, it is possible to establish the self-trapping effect in the nonexcited core, however the sufficient increase of the energy will trap the solitonic pulse already in the excited core, thus it prevents the cross-transfer. Therefore, the proper fiber and pulse parameters can provide a working point around which the slight change of the pulse energy governs the swap of the trapping effect between the two cores, i.e. high-contrast switching takes place. The initial dual-core asymmetry causes some modifications of the switching mechanism, because the increasing nonlinearity during the self-compression process can induce not only trapping in the proper core but also balancing of the dual-core asymmetry. Differentiating between the fast and slow fiber core determined by the effective refractive index, effective switching can take place only in the case of fast core excitation, because it can ensure asymmetry balancing by the Kerr nonlinearity. Based on this description, let us summarize the most important findings comparing the observed self-switching performance in the more asymmetric central DCU and less asymmetric bottom DCU. The lower asymmetry character was confirmed both by stronger fiber-length dependence of the ER at low pulse energies (first ER value in the table cells) caused by the coupling oscillations in the linear propagation regime and by the numerical analysis of the both DCUs. The difference between the fiber lengths where the maximal ER (27 mm) were registered and the minimal ER (13.7 mm) at low pulse energy determines the coupling length resulting in a value of 13.3 mm, which is in correspondence with both by the calculated and experimentally identified *L*_c_ in similar fiber types (Longobucco 2021).

In the case of the all DCUs only the excitation of one of the cores resulted in switching behavior, which finding confirms the concept of asymmetric couplers requiring excitation of the fast core for effective cross-transfer. The more asymmetric central DCU express lower switching energies at longer optimal fiber lengths to express high contrast switching. In this case, the asymmetry prevents the cross-transfer at initial pulse energies, thus, the soliton compression process is not disturbed by the coupling effect. As a result, the soliton-like pulse is compressed gradually in the excited core up to the distance where the balancing effect takes place. Afterwards, the cross-transfer requires maintaining of the pulse peak power at similar level along a certain distance, therefore, the soliton compression distance should be much longer than the coupling length. Fulfilling this condition ensures the balancing up to the point where the majority of energy is transferred. Thus, in the case of the higher asymmetry, a lower switching energy and longer fiber are expected in correspondence with the central DCU results, where the fiber length is about three times longer than the coupling length. In contrast, the lower asymmetry supports cross-transfer at shorter fiber lengths, as it is already accessible at lower peak powers during the self-compression process and starts at an earlier phase of the propagation. However, it requires higher pulse energies to trap it in the excited core without cross-transfer, which is necessary to establish high switching contrasts. These characteristic features resemble the results obtained in the case of bottom DCU excitation with lower asymmetry, and therefore exhibit modified switching regime.

Further, the lower asymmetry DCU can support more switching steps because the core of the trapped soliton can be alternated several times by subsequent increasing of the energy and shortening the compression distance (Nguyen et al. 2023). The higher asymmetry DCU cannot support similar number of switching steps, because the shorter soliton compression distances does not maintain the pulse peak power along propagation lengths necessary for the cross transfer. In summary, the shorter optimal lengths, higher switching energies and more switching steps in the case of the bottom unit confirm the theoretical predictions of the lower asymmetry effect on the self-switching performance. After the overview and theoretical evaluation of the main results we will present the improved switching performance identified in selected experimental conditions.

### Self-switching performance at higher level of dual-core asymmetry

**Fig. 2:**
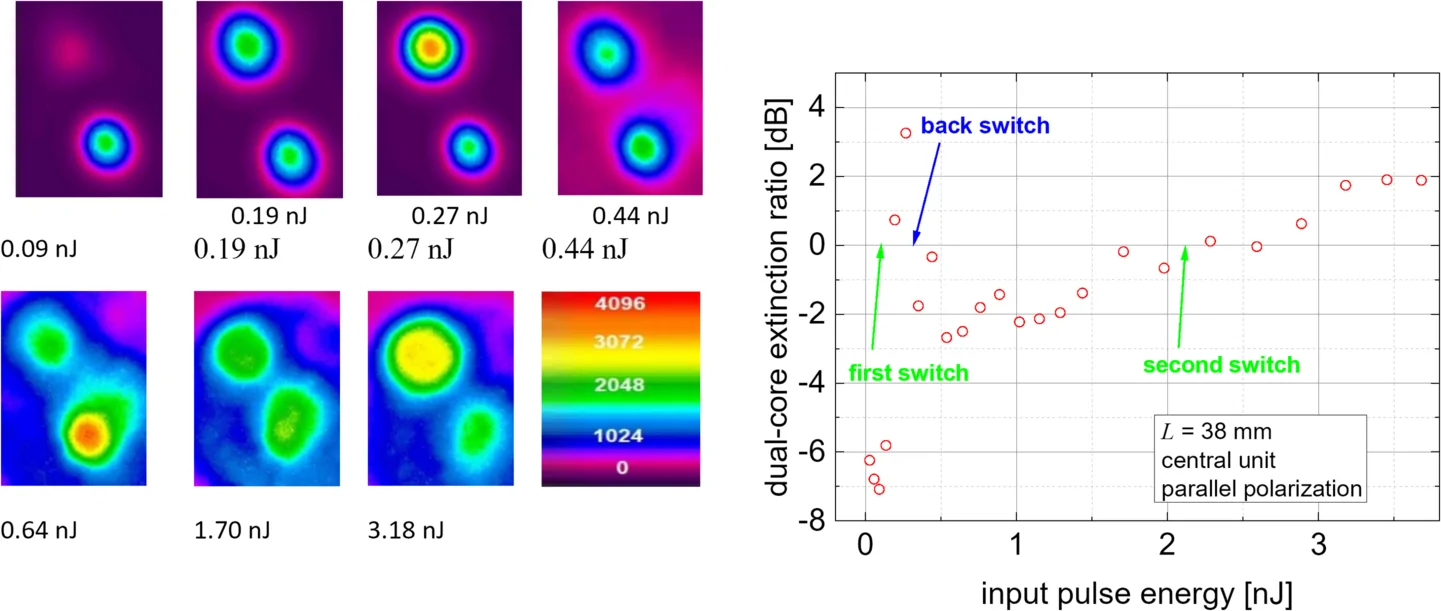
Central DCU at 38 mm fiber length and parallel polarization: **a** series of selected IR camera images of the output facet of the central DCU under increasing pulse energies expressing double switching behavior and **b** extinction ratio dependence on pulse energy based on processed camera images of the. The smooth dependence with threefold ER sign change with significant amplitude confirms the double switching behavior

The first registrations were performed at the starting 38 mm fiber length. Nonlinear switching was observed in the case of three units: left, central, and right for both orthogonal polarizations with remarkable switching contrasts (SC) above 15 dB in five cases. On the other hand, the switching symmetry, i.e. balance between the absolute values of ER_max_ and ER_min_, was at the most of the combinations poor and in the case of left parallel no dominance exchange was observed despite of the highest observed SC at this length. In contrast, the central parallel results express the lowest SC = 10.4 dB, however, at the best switching symmetry (3.3 dB vs. −7.1 dB) and lowest switching energy of 0.27 nJ among all combinations. The camera-based results yielding these values are presented in Fig.2. Due to the above listed switching parameters, the central parallel seemed to be the most promising for all-optical signal processing applications because of the proper level of propagation constant mismatch and fiber length. For example, in the case of optical router it is important that the device can operate at low power consumption level and that the switched output energy is comparable between the both output ports ensured by the symmetrical switching character. From the latter point of view, the performance presented in Fig. 2a is still not optimal because the output field intensity in the less dominant bottom core at 0.27 nJ energy is almost equal to the values registered in the case of its dominance at 0.09 nJ. It is worth mentioning the camera images above 0.5 nJ express some deviations from the single mode character, which is routinely ensured by the careful in-coupling optimization process at low energies. Such deformations are caused most probably by the nonlinear spectral broadening combined by the chromatic aberration of the output microscope objective. However, those don’t affect significantly the evaluation of the switching performance, which is based on integral estimation of the output energy considering the all camera pixels bearing information about the intensity spatial distribution.

Interesting double switching behavior is observable on the energy dependent series, which is well distinguishable in the graph of Fig. 2b expressing three consecutive changes of the ER sign. Such behavior is a confirmation of the solitonic switching character described in the previous section, because the self-trapping phenomenon allows more exchanges of the output port dominance. It is worth mentioning that the important advantage of the soliton self-trapping phenomenon is the localization of the pulse energy in one core for longer distances after the compression point due to the high peak power-induced asymmetry. Thus, if the first trapping effect takes place in the excited core after the first oscillation period, the subsequent shortening of the soliton compression point can establish trapping in the nonexcited and afterwards again in the excited core, resulting in double switching behavior. It was demonstrated several times by exciting similar fiber (Longobucco et al. 2020a, b; Longobucco et al. 2020a, b), however we present it now first time in C-band thanks to the proper dual-core symmetry found out among the high amount dual-core units and polarization orientations.

**Fig. 3:**
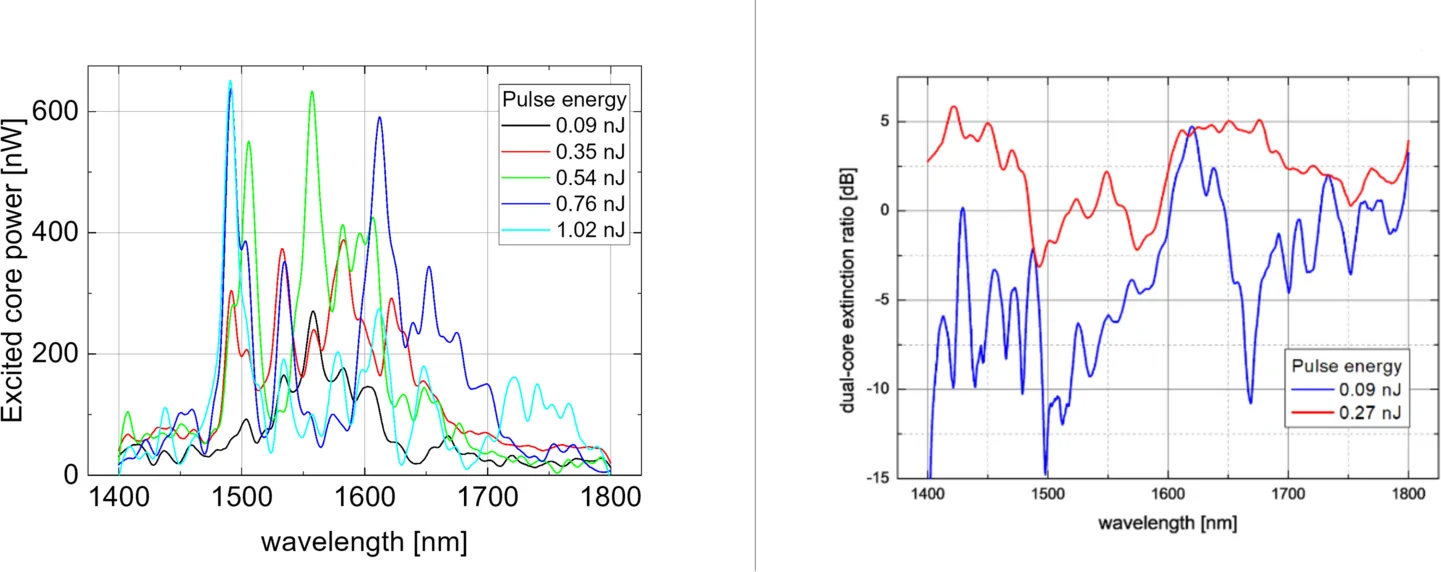
Spectral registrations in the case of central DCU at parallel polarization and 38 mm fiber length: **a** input energy dependence of the raw spectra registered from the excited core. **b** Spectrally resolved extinction ratio at two selected energies expressing broadband switching behavior

In Fig. 3. we present the spectral data registered at the same parameter combination. Panel (a) displays the raw spectra registered from the excited core expressing separated spectral features, especially above 0.4 nJ, which is common in the femtosecond solitonic propagation regime due to the soliton fission process. Up to an energy level of 0.54 nJ the spectra express nearly homogeneous broadening with dominance of spectral intensities around the central wavelength. Consequently, at this moderate nonlinear interaction regime one can suppose the preservation of the pulse integrity supporting the optical signal processing application. In contrast, the 0.74 and 1.02 nJ curves unveil serious pulse distortions, thus only the lower switching energies represent real application potential. Therefore, in panel (b) is shown the spectrally resolved dual-core extinction ratio in the case of the 0.09 and 0.27 nJ pulse energies, which represent switch from the nonexcited to the excited in correspondence with the camera results. The two spectra are situated nearly fully in the negative and positive half of the graph and express solid switching contrast around the central wavelength in an area of 1500–1600 nm approaching the level of 10 dB at the short wavelength part. Therefore, the spectral results confirm the dominance exchange observed by the camera and reveal the broadband switching character up to pulse energies of 0.54 nJ.

### Fiber length study in the case of best switching performances

**Fig. 4:**
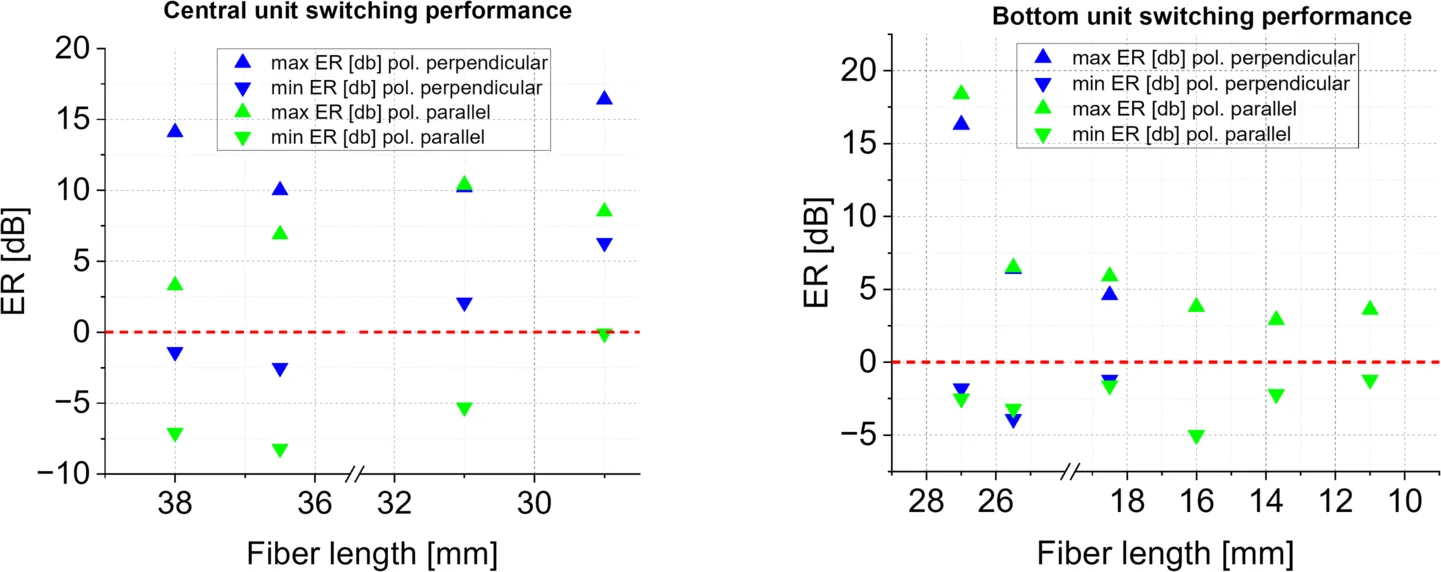
Fiber length dependence of maximum and minimum ER value in the case of the **a** central and **b** bottom DCU for the both orthogonal polarization directions

Similar registrations were conducted by successive shortening of the optical fiber, and two sequences of lengths with persistent nonlinear self-switching behavior were identified presented in Tables 1 and 2 related mainly to the central and bottom DCU, respectively. In the case of the former, dominance exchange was observed for fiber lengths in the range of 38–29 mm and the central parallel excitation case presented above was confirmed as the most advantageous. In the case of the latter, bottom parallel expressed the best switching parameters in range of 27–11 mm, however, both units express switching performance also for perpendicular polarization, however only at larger lengths. In Fig. 4 are presented the dependences of minimal and maximal ER on fiber length for (a) central and (b) bottom DCU. The two dependences express some common properties by comparing the two polarization directions. The parallel polarization brought better switching performances in terms of switching persistence and symmetry, and the switching in the case of perpendicular polarization is observable only in the longer half of the parallel polarization switching ranges, and consequently the length of the range is reduced. Another tendency of comparing the two polarization directions is the lower energy requirement of the best switching performance for the parallel polarization observed in the results of the both units. Based on our previous interpretation, the higher energy requirements can be explained by lower dual-core asymmetry in the case of the perpendicular polarizations. In the case of such an interpretation it could also be accompanied by shorter optimal length, however, this effect is not so clear comparing the two orthogonal polarizations. It can be caused by the fact that the polarization direction does not change only the propagation constant mismatch, however, the coupling length as well and the combination of these two effects determines the range of the switching lengths. The convincing evaluation of the polarization effect would require rigorous analysis of the femtosecond nonlinear propagation taking into consideration the all-linear fiber characteristics, which task is beyond the scope of this experimental work. Nevertheless, we can state that the parallel polarization enables better switching performance, most probably due to the better coupling between the cores when the electric field vector is oriented toward the connection line between the cores.

**Fig. 5:**
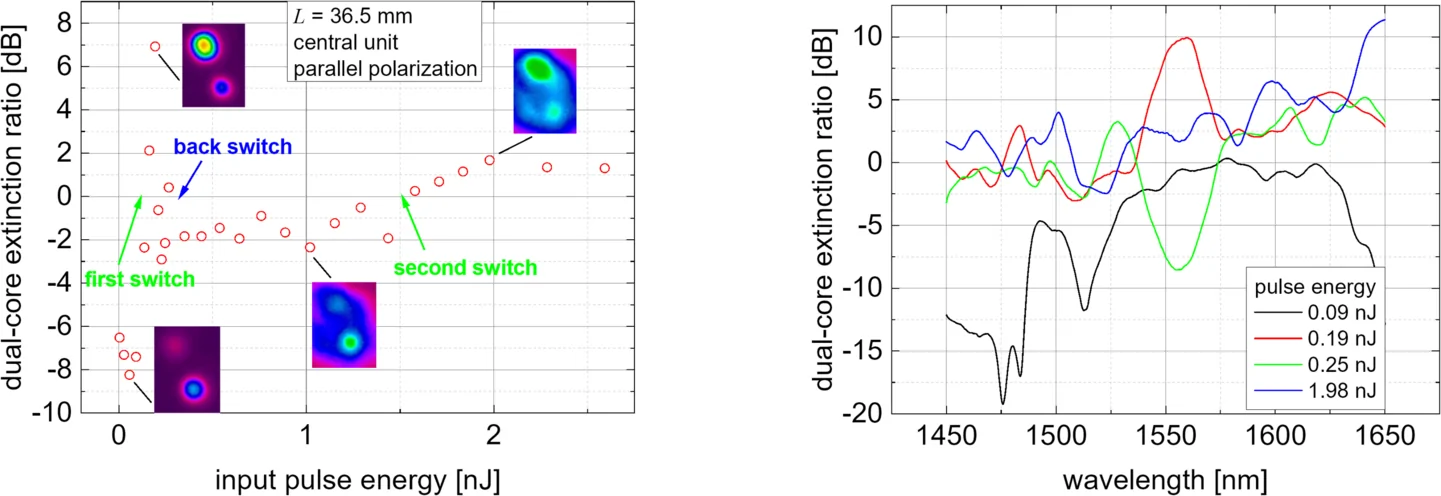
**a** Extinction ratio dependence on pulse energy based on processed camera images of the central DCU at 36.5 mm fiber length and parallel polarization. **b** Spectrally resolved extinction ratio dependence on the pulse energy registered under identical conditions

In the case of excitation of the central unit under parallel polarization, the optimal fiber length is 36.5 mm with ER dependence on input pulse energy presented in Fig.5a together with the camera images captured at energies causing extremal ER values. It expresses the best switching symmetry and lowest switching energy with parameters: −8.24 dB at 0.06 nJ and 6.92 dB at 0.19 nJ, and thus the switching contrast exceeds 15 dB. Furthermore, double switching behavior was observed at this set of parameters with subsequent steps: −2.35 dB at 1.02 nJ and 1.67 dB at 1.98 nJ. The energy dependence similarities in the case of 38 mm (Fig. 2b) and 36.5 mm (Fig. 5a) fiber lengths, are obvious. The same double-switching behavior is observable with very similar values of extremal switching energies and ERs. These results provide direct confirmation of the soliton self-trapping phenomena responsible for the studied switching performance. Beside the soliton compression point the pulse energy is trapped in the respective core without further cross-transfer along a certain distance. This is the reason for the almost identical double exchange of the dominance core at the output despite the different fiber lengths. Moreover, the high contrast switching was preserved also up to the 31 mm fiber length exhibiting the highest switching contrast (15.7 dB) and lowest switching energy (0.16 nJ) of the central parallel series. Based on these results, it is possible to estimate the soliton compression distance at the 0.16 nJ energy to be around 30 mm, enabling the high-contrast switching at slightly longer fiber lengths and causing sudden drop of the switching performance at shorter fiber lengths observable at 29 mm length. The same effect was found in the case of perpendicular polarization expressing a slightly longer soliton compression point localized in range 31–36 mm caused by the lower asymmetry (*δ* = 1360 m^−1^) disturbing more the nonlinear propagation by the coupling effect. The described dual-core field evolution character demonstrated in the C-band are in line with outcomes of experimental/theoretical study performed at 1700 nm (Nguyen et al. 2023). In that work the self-trapping mechanism was identified behind the double switching effect in the case of asymmetric couplers enabling the preservation of such advanced performance despite of change of the fiber length.

The spectral picture registered under the same conditions confirms also the improved performance at the 36.5 mm fiber length presented in Fig. 5b. Clear broadband switching behavior is visible when comparing the ER spectra at 0.09 and 0.19 nJ energies spanning along the entire spectral range of the pulses. Furthermore, the central spectral range is especially emphasized in the back switching step observed at 0.25 nJ energy expressing a switching contrast of about 18 dB at the central wavelength, directly in the C-band of the optical communication. The final switching to the non-excited core at 1.98 nJ energy a exhibits also broadband character with ER values localized almost entirely on the positive half of the graph.

**Fig. 6:**
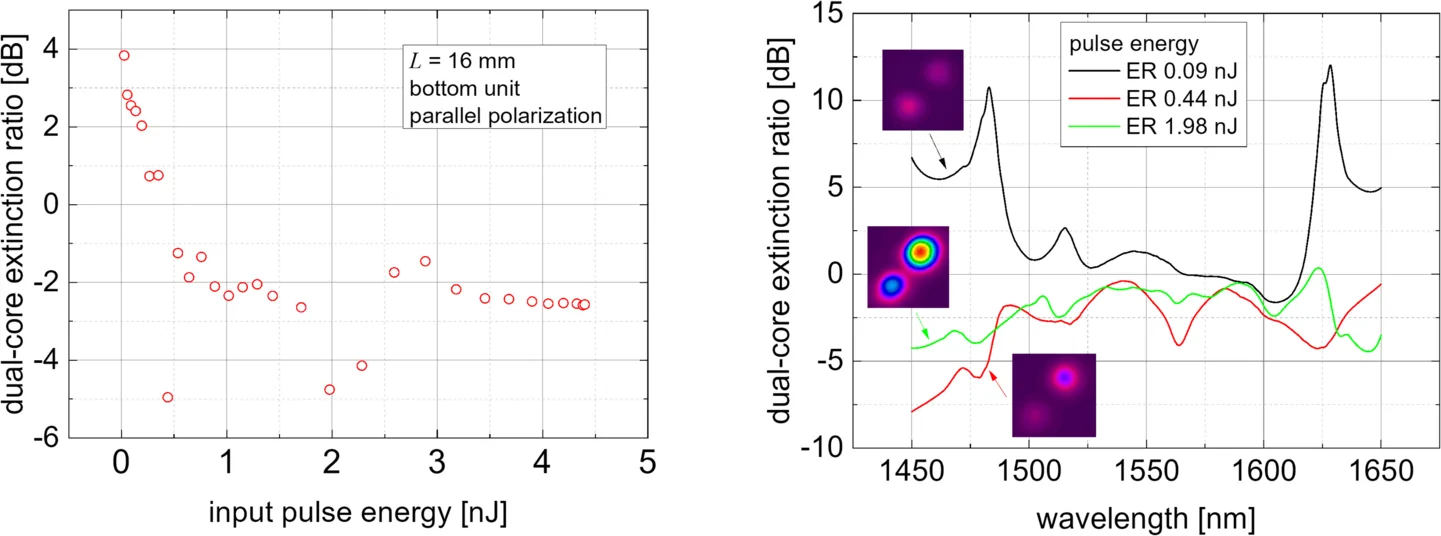
**a** Extinction ratio dependence on pulse energy based on processed camera images of the bottom DCU at 16 mm fiber length and parallel polarization. **b** Spectrally resolved extinction ratio dependence on the pulse energy under identical conditions

The bottom DCU in the case of parallel polarization direction indicates the lowest asymmetry estimated at the level of = 520 m^−1^ and enabled the longest fiber length range with switching performance and the shortest possible fiber lengths. Another characteristic indicating low asymmetry is the number of switching steps at the level of 24 in the range of fiber lengths of 27–18.5 mm. All of these positive properties were achieved at the cost of highest switching energies required for the shortening of the compression distance and eliminating of the reduction of the nonlinear effect by the coupling oscillations. Although applicable switching performance was observed at 16 mm length requiring only 0.44 nJ energy and expressing symmetrical switching character with contrast of 8.8 dB. In Fig.6a the energy dependence of the dual-core extinction ratio is presented expressing only one switching step, however, periodic character. The possible interpretation of these results is that the short fiber length does not allow the soliton compression localization between the two cores. When the first soliton compression effect is reached at the lowest possible energy (0.44 nJ) corresponding to the 16 mm length, the field is localized always in this nonexcited core even under further energy increase. Only the strength of the localization is modulated by the pulse energy reaching a second level (1.98 nJ) at a shorter distance, allowing self-trapping along the rest of the propagation length. This interpretation is supported by the finding that the switching is still possible at shorter fiber length at the second energy level in the few nanojoules energy range. It is worth mentioning that the dominance exchange between the cores was observed up to 11 mm at 2.28 nJ switching energy, which represents the shortest demonstrated fiber length in the case of self-switching approach in similar DCFs. The spectral results in Fig. 6b confirm the applicable switching performance under the optimal 16-mm length. The comparison of the ER spectra between switching energy pairs 0.09/0.44 nJ and 0.09/1.98 nJ express the broadband character across the 1450–1650 nm range. Furthermore, in the case of the 0.09/0.44 nJ pair, enhanced cross-transfer is visible right around the central wavelengths in correspondence with the higher switching contrast registered by camera.

## Conclusions

In this work, we systematically investigated highcontrast alloptical selfswitching of femtosecond pulses using a specialty softglass multicore fiber composed of five noninteracting dualcore units. By exciting all fiber cores in both principal orthogonal polarization axes and exploring fiber lengths from 11 to 38 mm, we revealed a broad ensemble of switching behaviors that surpass previously reported results. A key advantage of the studied structure was the ability to compare dualcore units with different levels of optical asymmetry, corresponding to propagationconstant mismatches between 500 and 2100 m⁻^1^ at 1560 nm. In Table 3 we list all 4 numerically determined mismatches in the case of the two analyzed units for the both orthogonal polarization directions together with the effective coupling coefficient. Further, the table contains the experimental switching parameters as contrast, energy and steps expressing convincing correlation between the asymmetry level and switching quality. All switching parameters rise monotonically with decreasing asymmetry.

**Table 3:** Relation between the numerically determined mismatch and effective coupling coefficient in the case of the two analyzed units for the both orthogonal polarization directions to the experimental switching parameters as contrast, energy and steps

Dual-core unit	δ [m⁻^1^]	ϰ eff. [m⁻^1^]	Sw. contrast	Sw. energy	Sw. steps
Central par.	2100	2190	15.1 dB	0.19 nJ	3
Central perp.	1360	1410	15.5 dB	0.54 nJ	3
Bottom perp.	885	1145	18.1 dB	1.15 nJ	4
Bottom par.	520	725	20.9 dB	1.15 nJ	4

The observed switching dynamics were interpreted in terms of controllable self-trapping of highorder solitons between the two cores, modified by the imposed asymmetry. The combined action of soliton selfcompression and nonlinear asymmetry balancing enabled the lowest switching energy reported to date, achieved in the unit with the highest mismatch (δ = 2100 m⁻^1^). The measured switching energy of 0.19 nJ represents a significant improvement over earlier works, reaching the sub100pJ regime when accounting for the 50% incoupling efficiency (Longobucco et al. 2021; Nguyen et al. 2020). At a fiber length of 36.5 mm, the switching contrast exhibited a nearly symmetric response (6.9 dB vs. –8.2 dB).

In contrast, the lowest mismatch (δ ≈ 520 m⁻^1^) enabled high-contrast switching at the shortest device length of 16 mm, although at a higher switching energy of 0.44 nJ. This level of asymmetry allows the dominant core to change with propagation distance under linear conditions, leading to a qualitatively different non-linear switching regime. While a higher energy is required to suppress linear coupling oscillations, the same higher energy also shortens the soliton-compression distance, enabling compact high-contrast switching. The mentioned mutual effects resulted in optimized conditions that reduced the device length to 16 mm and increased the number of switching steps to 4 at a fiber length of 25.5 mm, performance levels not previously achieved in comparable studies (Longobucco et al. 2021; Nguyen et al. 2020). Spectral measurements under all effective switching conditions confirmed broadband high-contrast operation without significant pulse distortion, demonstrating the strong application potential of the proposed approach under the improved conditions. It is important to stress that the various switching regimes are available utilizing the same piece of fiber, enabling also the low switching energy or short fiber length advantage depending on the requirement of the application.

## Data Availability

The datasets generated and analyzed during the current study are available from the corresponding author on reasonable request.
